# Rectal gonorrhoea and chlamydia among men who have sex with men in coastal Kenya

**DOI:** 10.12688/wellcomeopenres.15217.4

**Published:** 2020-06-04

**Authors:** Caroline J. Ngetsa, Marc W. Heymann, Alex Thiong'o, Elizabeth Wahome, John Mwambi, Clara Karani, Nelson C. Menza, Grace Mwashigadi, Margaret W. Muturi, Susan M. Graham, Peter M. Mugo, Eduard J. Sanders

**Affiliations:** 1 Department of Bioscience, KEMRI-Wellcome Trust Research Programme, Kilifi, Kenya; 2 Department of Medicine, Barts and The London NHS Trust, London, E11BB, UK; 3 Department of Medical Laboratory Sciences, Kenyatta University, Nairobi, Kenya; 4 Department of Medicine, University of Washington, Seattle, WA, USA; 5 Department of Global Health, University of Washington, Seattle, WA, USA; 6 Department of Epidemiology, University of Washington, Seattle, WA, USA; 7 Nuffield Department of Medicine, University of Oxford, Headington, UK

**Keywords:** Chlamydia, Gonorrhoea, Men Who Have Sex With Men, Kenya, Cefixime, Azithromycin, Antimicrobial Susceptibility

## Abstract

**Background:** Men who have sex with men (MSM) have a higher prevalence of *Chlamydia trachomatis* (CT) and *Neisseria gonorrhoeae* (NG) infections compared to the rest of the population, often remaining undiagnosed. In Kenya, prevalence of rectal CT and NG infection and NG antimicrobial sensitivity are poorly described.

**Methods:** MSM who reported receptive anal intercourse (RAI) were recruited from an ongoing human immunodeficiency virus acquisition and treatment study in coastal Kenya in 2016-2017. Rectal swabs were collected at two time points 6 months apart to estimate prevalence and incidence of CT/NG infection using a molecular point-of-care assay. Participants positive for CT or NG were treated according to national guidelines. NG culture and antimicrobial susceptibility testing was performed. Participant and risk behaviour characteristics were collected and association with baseline CT/NG prevalence assessed by multivariable regression analysis.

**Results:** Prevalence of CT/NG in 104 MSM was 21.2% (CT 13.5%, NG 9.6%, dual infection 1.9%) at baseline and 25.9% in 81 MSM at follow-up (CT 14.8%, NG 14.8%, dual infection 3.7%). CT/NG incidence was estimated at 53.0 (95% CI, 34.5-81.3) per 100 person-years. Most CT/NG positive participants were asymptomatic: 95.5% at baseline and 100% at follow-up. CT/NG infection was associated with being paid for sex [adjusted odds ratio (aOR)=6.2, 95% CI (1.7-22.9)] and being in formal employment [aOR=7.5, 95% CI (1.1-49.2)]. Six NG isolates were obtained at follow-up; all were susceptible to ceftriaxone and cefixime and all were resistant to penicillin, tetracycline and ciprofloxacin.

**Conclusions:** There is a high prevalence and incidence of asymptomatic rectal CT and NG in MSM reporting RAI in coastal Kenya. MSM who were paid for sex or had formal employment were more likely to be infected with CT/NG suggesting increased risk behaviour during transactional sex. Antimicrobial susceptibility results suggest that current antibiotic choices in Kenya are appropriate for NG treatment.

## Introduction

*Chlamydia trachomatis (CT)* and *Neisseria gonorrhoeae (NG)* infections are curable sexually transmitted infections (STIs) that can have distressing symptoms and complications, though a large proportion remains asymptomatic ^1^. Global estimates of prevalence of genitourinary CT and NG infections from 2009 to 2016 were 2.7% and 0.7%, respectively, amongst men aged 15–49 years old ^2^. Men who have sex with men (MSM) are a key population with regards to transmission of STIs. Prevalence of rectal CT and NG infections has ranged as high as 21.7%-27.2% and 13.8%-25.4%, respectively in Italian and Thai MSM communities ^3, 4^. As a traditionally highly stigmatized population, the prevalence and incidence of STIs in MSM in sub-Saharan Africa has only been studied since 2005 ^5, 6^. Prevalence of rectal CT and NG has been estimated at 8% and 8%, respectively, in South African MSM above the age 18 ^7^, 13.5% and 23.3%, respectively, in adult Nigerian MSM ^8^ and 28.2% and 29.5%, respectively, in adult Tanzanian MSM (median age 23) ^9^. In Kenya, we have estimated the prevalence of rectal CT and/or NG to be 11.6% in a small sample of 43 human immunodeficiency virus (HIV)-negative adult MSM in the coastal region ^10^ and 5.2% in HIV-positive and HIV-negative MSM over the age of 18 in the western part of the country ^11^. In MSM, younger age, sex with men only (as opposed to sex with both men and women), transactional sexual intercourse, unprotected anal intercourse, and being HIV positive have been found to be associated with rectal CT/NG ^11– 14^.

Over the years, the diagnosis and treatment of CT and NG infection has changed as a result of evolving technology and the development of antimicrobial resistance (AMR). Diagnosis is based on a combination of clinical suspicion, such as symptoms and signs suggestive of urethritis or proctitis, and laboratory investigations ^15, 16^. Nucleic acid amplification testing (NAAT), both highly sensitive and specific in detecting CT and NG, has superseded the use of cultures for the detection of infection where available ^16– 18^. In addition, with the advent of commercial point-of-care (POC) NAAT tests such as the GeneXpert® CT/NG assay (Cepheid), results can be obtained much more rapidly (90 minutes with the GeneXpert®) ^15, 17, 18^. The provision of same-day results is of particular importance in treating the MSM population, which is less likely to attend healthcare services ^6^. In the context of NG infection, however, bacterial culture is still recommended to enable detection of AMR ^19^.

Globally, AMR is a major threat to public health and resistance to the key antibiotic classes has been detected ^20^. In Kenya, plasmid-mediated resistance of NG to penicillin and tetracycline has been shown to be more prevalent than in other countries and is likely related to extensive use of doxycycline ^21^. The World Health Organization (WHO) currently recommends treating CT with either azithromycin or doxycycline ^22^. Where sensitivity is unknown, the WHO recommends treating NG with ceftriaxone or cefixime combined with azithromycin. In cases where sensitivity is known, a single agent can be used ^23^.

The WHO has recommended syndromic treatment of STIs as an answer to the low health-seeking behaviour and continuity of care typically seen in developing countries ^24, 25^. In Kenya, first-line recommended treatment for proctitis is cefixime or ceftriaxone and azithromycin ^26^. Given higher prevalence amongst MSM, the WHO recommends presumptive treatment of MSM reporting unprotected receptive anal intercourse (RAI) in the previous six months and either a partner with an STI or multiple partners ^25^. Sensitivity of this approach has been estimated at 74.1% with a specificity of 45.8% in MSM in coastal Kenya ^27^. This study was performed to estimate the prevalence of asymptomatic CT and NG infections in MSM reporting RAI and to assess the susceptibility of NG to commonly used antimicrobials.

## Methods

### Study design

The study was conducted between April 2016 and January 2017 at the Kenya Medical Research Institute (KEMRI) Mtwapa Clinic, located in a town popular for its beach tourism and nightlife. A total of 104 MSM of 174 eligible participants were recruited from an ongoing cohort study of high-risk MSM ^28^. Participants not enrolled were more likely to report sex with men exclusively, transactional sex, RAI, group sex, and any unprotected sex (data in supplemental material). Men reporting RAI in the previous 6 months and aged between 18–49 years old were included for analysis. MSM were tested for CT/NG at baseline and at a follow-up visit 6 months later. Participants who tested positive were treated accordingly; CT infection was treated with azithromycin (1 g stat dose) and doxycycline (100 mg twice a day for 7 days), NG infection with cefixime (400 mg stat dose) and azithromycin (1 g stat dose) and dual infection treated with triple therapy (cefixime, azithromycin and doxycycline). A clinician observed the participant taking cefixime, azithromycin and the first dose of doxycycline.

### Collection of participant characteristics

At baseline, counsellors conducted personal interviews with the participants. Men were considered to have sex with men exclusively (MSME) as opposed to both men and women (MSMW) based on answers they had given in previous clinic visits; if they had never reported having sex with women, they were considered MSME. Participants were asked if they had received money for or paid for sex in the 3 months prior to CT/NG sample collection. Condom use and number of sexual partners was also assessed. Clinicians recorded participant symptoms at the visit, and a participant was considered symptomatic of proctitis if he complained of rectal discharge, pain or an ulcer in the perianal region.

### Sample collection and antimicrobial susceptibility testing for NG

At baseline and follow-up, a trained clinician collected rectal swabs in all patients using a proctoscope and dry GeneXpert® CT/NG swabs. The first swab was used to detect CT and NG infection by NAAT with the GeneXpert® CT/NG Assay (Cepheid AB, Sweden), and the second was inoculated on Thayer Martin Modified agar. Inoculated plates were transported to a reference laboratory 40 km away at the end of each day for culture and antimicrobial susceptibility testing of NG ^29^. A case of NG or CT infection was defined as being a positive result on the GeneXpert® CT/NG assay.

The disc diffusion test was used to assess antimicrobial susceptibility of NG to ceftriaxone, penicillin and tetracycline ^30^. The Etest was performed to obtain minimum inhibitory concentrations (MIC) for cefixime. ciprofloxacin and penicillin; cut-off values, as follows, were obtained from the Clinical and Laboratory Standards Institute (CLSI) ^29^. For cefixime, a MIC ≤0.25 μg/ml indicated susceptibility. For ciprofloxacin, a MIC of ≤0.06 μg/ml indicated susceptibility, 0.12-0.5 μg/ml intermediate susceptibility, and ≥1 μg/ml resistance, while for penicillin, a MIC of ≤0.06 μg/ml indicated susceptibility, 0.12-1.0 μg/ml intermediate susceptibility, and ≥2 μg/ml resistance.

### Data analysis

Data were analysed using Stata 13.0 (StataCorp LP, College Station, TX). Baseline participant characteristics were described. Prevalence of NG and CT at baseline and follow-up was estimated, as were incidence rates of each infection. Univariable linear regression was used to estimate association between participant characteristics and prevalence of CT / NG infection at baseline. Any variable with a p-value of *p<0.2* was used in a multivariable regression analysis to determine adjusted odds ratios (aOR). Age was used a-priori in the multivariable regression, given that the population at the clinic is principally under 25 years of age and that STIs are more prevalent in younger age groups ^13, 31^.

### Ethical approval

Ethical approval (protocol numbers 894 and 1224) was obtained from the KEMRI/Scientific and Ethics Review Unit (KEMRI/SERU). Written informed consent was obtained from every participant.

## Results

### Participant characteristics

Between April and July 2016, 104 participants were recruited and tested. Participant characteristics are described in full in Table 1. Approximately half (55.8%) of those recruited were between the ages of 25–34, approximately nine out of ten (86.5%) were never married, and approximately half (49.0%) received secondary education only. Half the participants (51.0%) were Christian one quarter were Muslim (26.0%) and 23.1% were either not religious or of a different religious background. One in four (25.0%) were unemployed, with the rest either self-employed (57.7%) or in formal employment (17.3%). One in four (25.0%) of the participants reported unprotected sex in the week preceding testing; with a similar number indicating they had sex with men exclusively (26.9%). Half the participants had been paid for sex in the 3 months prior to the study. Just over one in three (34.6%) MSM were HIV positive.

**Table 1.** Risk factors associated with *Chlamydia trachomatis* (CT) and/or *Neisseria gonorrhoeae* (NG) infection in 104 men who have sex with men (MSM) at baseline.

Variable	All, n (% of total)	CT/NG, n (%)	OR, (95% CI)	*p* value	AOR (95% CI)	*p* value
Age (years) *						
18–24	36 (34.6)	10 (27.8)	0.9 (0.2-4.2)	0.890	1.2 (0.2 – 8.5)	0.883
25–34	58 (55.8)	9 (15.5)	0.4 (0.1-2.0)	0.277	0.8 (0.1 – 5.7)	0.853
35+	10 (9.6)	3 (30.0%)	**Reference**	-	**Reference**	-
**Education**						
Primary/none	43 (41.4)	9 (20.9)	**Reference**	-		
Secondary	51 (49.0)	13 (25.5)	1.3 (0.5-3.4)	0.603		
Higher/tertiary	10 (9.6)	0 (0.0)	*n/a ^δ^*	*n/a ^δ^*		
**Marital status**						
Never married	90 (86.5)	19 (21.1)	**Reference**	-		
Ever married	14 (13.5)	3 (21.4)	1.0 (0.3-4.0)	0.978		
**Religion**						
Christian	53 (51.0)	13 (24.5)	**Reference**	-		
Muslim	27 (26.0)	6 (22.2)	0.9 (0.3-2.6)	0.819		
Other/None	24 (23.1)	3 (12.5)	0.4 (0.1-1.7)	0.237		
**Employment ^‡^**						
None	26 (25.0)	3 (11.5)	**Reference**		**Reference**	-
Self	60 (57.7)	13 (21.7)	2.1 (0.5-8.2)	0.275	1.8 (0.4-7.8)	0.416
Formal	18 (17.3)	6 (33.3)	3.8 (0.8-18.1)	0.090	7.5 (1.1-49.2)	**0.036**
**Sex with** ^‡^						
MSMW	76 (73.1)	13 (17.1)	**Reference**	-	**Reference**	-
MSME	28 (26.9)	9 (32.1)	2.3 (0.9-6.2)	0.101	2.3 (0.7-7.1)	0.159
**Received payment for sex in past 3 months** ^‡^						
No	52 (50.0)	5 (9.6)	**Reference**	-	**Reference**	-
Yes	52 (50.0)	17 (32.7)	4.6 (1.5-13.6)	**0.006**	6.2 (1.7-22.9)	**0.006**
**Paid for sex in past 3 months**						
No	97 (93.3)	21 (21.7)	**Reference**	-		
Yes	7 (6.7)	1 (14.3)	0.6 (0.1-5.3)	0.648		
**Alcohol use in past month**						
No	62 (59.6)	12 (19.4)	**Reference**	-		
Yes	42 (40.4)	10 (23.8)	1.3 (0.5-3.4)	0.586		
**Sexual exposure and condom use in past** **week**						
No activity	34 (32.7)	6 (17.7)	**Reference**	**-**		
All protected	44 (42.3)	8 (18.2)	1.0 (0.3-3.3)	0.951		
Any unprotected	26 (25.0)	8 (30.8)	2.1 (0.6-7.0)	0.238		
**Condom use for anal sex in past 3 months** ^†^						
No	75 (72.1)	18 (24.0)	**Reference**	-		
Yes	26 (25.0)	4 (15.4)	0.6 (0.2-1.9)	0.363		
**Total sex partners in past month**						
None	15 (14.4)	3 (20.0)	**Reference**	-		
One	21 (20.2)	2 (9.5)	0.4 (0.1-2.9)	0.380		
Two or more	68 (65.4)	17 (25.0)	1.3 (0.3-5.3)	0.683		
**HIV status**						
Negative	68 (65.4)	15 (22.1)	**Reference**	-		
Positive	36 (34.6)	7 (19.4)	0.9 (0.3-2.3)	0.756		

*Included in the multivariable analysis priori. ‡Only factors significant at *p*≤0.2 in the univariable analysis were included in the multivariable model. †3 participants did not report anal sex in the last 3 months but reported within the past 6 months. MSME, MSM exclusively; MSMW, MSM and women. OR, odds ratio; aOR, adjusted odds ratio; CI, confidence interval.

### Prevalence of CT/NG

At baseline, 22 (21.2%, 95% CI 13.8-30.3%) of the 104 MSM who took part in the study tested positive for CT and/or NG; 10 had NG (9.6%) and 14 had CT (13.5%)- 2 (1.9%) had dual infection. Only one participant reported symptoms whilst the rest (95.5%) were asymptomatic. The symptomatic participant was one of the two participants co-infected with both CT and NG. At baseline, none of the 104 swabs taken for culture, including those from the ten GeneXpert® NG positive participants, grew NG.

The prevalence of CT and/or NG infection was associated with receiving payment for sex [adjusted odds ratio (aOR)=6.2, 95% CI (1.7-22.9)] and being in formal employment [aOR=7.5, 95% CI (1.1-49.2)] on multivariable logistic regression ( Table 1), in a model controlling for age, having sex with men only or both men and women (MSME or MSMW, respectively), employment status and receiving payment for sex in the previous three months. MSME was also associated with greater odds of prevalent CT/NG, though this did not reach statistical significance [aOR=2.7, 95% CI (0.7-7.1)].

Of the 104 participants included in the baseline analysis, 20 (19.2%) participants were lost to follow-up; 3 (2.9%) were missing a test at 6 months. Baseline CT/NG prevalence in these 23 participants was 31.8% (30.4%) compared to 18.5% in those who attended follow-up, although this difference was not statistically significant (p=0.217). Twenty-one (25.9%) of 81 participants at follow-up tested positive for CT and/or NG infection; 12 (14.8%) participants had CT, 12 (14.8%) had NG, and 3 (3.7%) had dual infection. Five of the twenty-one (23.8%) cases of CT or NG that tested positive for CT or NG at follow-up were also positive at baseline; 5 had CT and 1 NG (1 CT and NG co-infection). All 21 follow-up participants were asymptomatic. NG was isolated from 50% (six) of twelve GeneXpert® NG positive follow-up participants.

### Incidence of CT/NG

The 81 MSM who contributed data towards incidence calculation were followed up for a median of 5.7 months (interquartile range, 5.4–6.2 months), which amounts to a total follow-up time of 39.6 person-years. Overall, 21 MSM tested positive for CT or NG for an incidence rate of 53.0 (95% CI, 34.5-81.3) per 100 person-years ( Table 2). Of these, 12 tested positive for CT [incidence rate of 30.0, 95% CI (17.2-53.3)] including 5 who tested positive for CT at baseline [incidence rate of 109.4, 95% CI (45.5-262.7)] and 7 who tested negative for CT at baseline [incidence rate of 20.0, 95% CI (9.5-41.9)], p=0.009. From the 21 patients who tested positive for CT or NG at follow-up, 12 were positive for NG [incidence rate of 30.0, 95% CI (17.2-53.3)], including 1 who tested positive for NG at baseline [incidence rate of 25.5, 95% CI (3.6-181.1)] and 11 who tested negative for NG at baseline [30.8 (17.1-55.6)], p=0.950.

**Table 2.** Incidence of rectal CT and/or NG infections among 81 MSM, Mtwapa, Kenya.

Participants at baseline and follow up, N=81	Cases/person-years	Incidence per 100 person-years (95% CI)	P-value
**Any CT/NG**	21/39.6	53.0 (34.5-81.3)	n/a
Infection at baseline No, N=66 Yes, N=15			
	15/32.1	46.7 (28.2-77.5)	-
	6/7.5	79.8 (35.9-177.6)	0.281
**Any CT**	12/39.6	30.3 (17.2-53.3)	n/a
Infection at baseline No, N=72 Yes, N=9			
	7/35.1	20.0 (9.5-41.9)	-
	5/4.6	109.4 (45.5-262.7)	0.009
**Any NG**	12/39.6	30.3 (17.2-53.3)	n/a
Infection at baseline No, N=73 Yes, N=8			
	11/35.7	30.8 (17.1-55.6)	-
	1/3.9	25.5 (3.6-181.1)	0.950

CT: *Chlamydia trachomatis*; NG: *Neisseria gonorrhoeae;* CI: *Confidence interval*

### Antimicrobial susceptibility of *N. gonorrhoeae*

Out of the 185 cultures performed (104 at baseline, 81 at follow-up), only six (3.2%) isolated NG; all six had been detected by the GeneXpert® NAAT and were isolated from follow-up samples. All 6 NG isolates were found to be resistant to tetracycline and ciprofloxacin. Of the 6, 2 were resistant, 3 had intermediate susceptibility and 1 was susceptible to penicillin. All 6 were sensitive to ceftriaxone and cefixime ( Table 3).

**Table 3.** Antimicrobial susceptibility of rectal *Neisseria gonorrhoeae* isolates.

	Disc diffusion (Inhibitory zone in mm)			E Test (MIC in μg/ml)		
Isolate	Penicillin ^a^	Ceftriaxone ^b^	Tetracycline ^c^	Penicillin ^d^	Cefixime ^e^	Ciprofloxacin ^f^
**1**	12	45	14	32	0.016	4
**2**	6	36	18	8	0.016	3
**3**	39	45	13	0.19	0.016	16
**4**	34	40	14	0.25	0.016	4
**5**	38	45	19	0.19	0.016	2
**6**	37	45	16	0.024	0.016	4

^a:^ Zone ≥47mm indicates susceptibility, 27-46 mm indicates intermediate susceptibility and ≤26mm resistance. ^b:^ Zone ≥35mm indicates susceptibility. ^c:^ Zone ≥38mm indicates susceptibility, 31–37mm intermediate susceptibility, ≤30mm resistance. ^d:^ MIC ≤0.06μg/ml indicates susceptibility, 0.12-1 μg/ml intermediate susceptibility and ≥2μg/ml resistance. ^e:^ MIC ≤0.25μg/ml indicates susceptibility ^f:^ MIC of ≤0.06μg/ml indicates susceptibility, 0.12-0.5μg/ml intermediate susceptibility, and ≥1μg/ml resistance

## Discussion

This study assessed the prevalence and incidence of rectal CT and NG in 104 MSM, identifying one in five participants at baseline and one in four participants at follow-up who were infected with either or both infections, and an estimated CT/NG incidence of 53.0 per 100 person years. Similarly high prevalence and incidence of rectal CT/NG infections were reported from other parts of sub-Saharan Africa ^7– 8, 11^. Prevalence at baseline was statistically associated with receiving money for sexual intercourse, a four-fold increase in odds, which is consistent with the findings from previous studies ^7, 12, 32^. Men who engage in transactional sex may have increased vulnerability to STIs, which is compounded by an increased burden of psychosocial morbidities, such as stigmatization and discrimination ^32^. Being in formal employment was also associated with prevalent infection with CT/NG, possibly indicating that those with stable employment and, presumably, financial means engage in more transactional sexual activity; although there was no statistically significant association between paying for sex and rectal CT/NG prevalence.

The WHO recommends that CT/NG infections should be detected and treated in a timely manner, by either presumptive or syndromic treatment ^22, 23, 25^. This is particularly important as these infections can remain undiagnosed for a long time unless routinely screened for, and thus be easily transmitted to other sexual partners. In this study, 95.5% of infections at baseline and 100% of infections at follow-up were asymptomatic. There are more asymptomatic cases of anorectal CT/NG reported in this study compared to other studies on MSM from the USA (58% asymptomatic CT, and 69% asymptomatic NG) ^33^ and Kenya (58.3% asymptomatic CT/NG infection) ^11^. Larger research studies are needed to confirm these findings. Nevertheless, this does support the need for more structured screening programmes to detect asymptomatic infections in developing countries, particularly in high-risk populations such as MSM ^34, 35^.

No NG was isolated from baseline rectal swabs in GeneXpert® NG-positive participants. At follow-up, six NG isolates were successfully cultured from twelve GeneXpert® NG positive participants. The only methodological differences at follow-up was prior notification of the laboratory at the reference centre 40 km away that a participant was positive on the GeneXpert® assay and one extra day of incubation to give more time to the scanty colonies to grow. It is well recognized that test methodology and technical expertise have an impact on the positivity rates of samples. Hence, antimicrobial susceptibility testing may be more reliably evaluated using rapid molecular POC tests in the future ^36^. The low yield in this case may also be due to the practicalities of having to transport the culture plates to the reference laboratory and the order of swab collections. As the culture swab was taken last, the bacterial yield may have been insufficient to result in a positive culture.

Regardless of yield, antimicrobial susceptibility testing revealed that all cultured NG isolates were sensitive to cefixime (first line treatment for NG) and ceftriaxone, and that all isolates were resistant to tetracycline and ciprofloxacin ( Table 3). Though azithromycin sensitivity was not assessed and baseline cultures were negative, the susceptibility of NG to cefixime at follow-up suggests that all treated participants were cured. Previous susceptibility studies of NG have shown that AMR is rising globally, and is a very real threat to healthcare systems, particularly for those caring for high-risk populations ^20, 21, 37, 38^. Genomic studies have demonstrated that resistant NG strains are transmitted between MSM partners ^37^. This information calls for careful treatment of NG, and for routine antimicrobial susceptibility testing to be performed. With the given WHO recommendations of syndromic and presumptive treatment, it is possible that AMR may spread further as an increasing amount of studies highlight the ongoing high rates of resistant NG in MSM. It is therefore critical that future studies systematically review all existing data on prevalence, incidence and gonococcal AMR, and that global guidelines on STI treatment in MSM and other key populations are updated to take into account AMR and POC testing ^22, 23^.

Approximately a third of participants in this study (34.6%) were HIV positive, though this was not statistically associated with CT/NG prevalence. HIV infection has been found to be higher in participants with an underlying STI, presumably facilitated by inflammation of mucosal epithelium ^39, 40^. It is therefore critical to rapidly detect and treat STIs in order to minimize future HIV-related epidemics in the MSM populations ^40^. Several studies have looked at the association between pre-exposure prophylaxis (PrEP) and the incidence of STIs: these studies demonstrated increased rates of CT/NG infections with PrEP use- potentially related to increased screening for STIs- increased condomless sexual intercourse and decreased serosorting between sexual contacts ^41, 42^. Several months after this study concluded, HIV-negative MSM in the same larger cohort started receiving PrEP; it will be interesting to assess the impact this has on CT/NG infection rates in this population.

This study has several limitations. First off, the sample size was small, rendering statistical analysis and interpretation of results for subgroups difficult. Due to the small sample size, we combined CT/NG infections to improve statistical power. As a result, we are unable to comment on predictors of CT and or NG separately. A large number of eligible patients were not enrolled in the study, and from brief comparison those patients had higher reports of sexual risk behavior compared to enrolled participants (see supplemental data). Therefore, our CT and NG prevalence and incidence estimates may be underestimates of a true population prevalence and incidence. Additionally, though all cases of CT and NG were treated at baseline, a test of cure was not performed: it is not possible for us to ascertain whether all cases at follow-up were new cases (reinfection) or whether some may have been persistent cases. Furthermore, our study did not assess azithromycin resistance. Any future studies should assess this given the frequent use of azithromycin in syndromic treatment of rectal infections. Finally, the small yield of NG cultures highlights the difficulty of generalizing resistance data, and this calls for larger studies of antimicrobial resistance in Kenya.

## Conclusion

The prevalence of rectal CT/NG infections in MSM who report RAI in coastal Kenya is estimated at 21.2%, and the incidence rate at 53.0 per 100 person years. Baseline infections were associated with transactional sex and formal employment. All but one of the participants who tested positive for CT/NG infection were asymptomatic, supporting the use of a presumptive treatment approach. Gonococcal AMR is of serious concern, and thus there is a need for continued surveillance of NG antimicrobial susceptibility and for sharing of AMR data between research groups studying NG in Kenya.

## Data availability

### Underlying data

Figshare. Data Sharing Excel.xlsx. https://doi.org/10.6084/m9.figshare.7735001.v1 ^43^. This file contains infection status for each participant, alongside details of risk factors and de-identified demographic information

Figshare.Data_Sharing_Worksheet_CT_NG.xlsx. https://doi.org/10.6084/m9.figshare.9896396.v1 ^44^. This file contains information regarding variable names for above dataset.

### Extended data

Figshare.Comparison_Of_MSM_Recruited_Versus_not_Recruited.txt https://doi.org/10.6084/m9.figshare.11847099.v1 ^45^. This file contains supplemental data comparing characteristics of eligible patients not recruited versus those that were.

Data are available under the terms of the Creative Commons Attribution 4.0 International license (CC-BY 4.0).
