# Supplementary material for: Rectal gonorrhoea and chlamydia among men who have sex with men in coastal Kenya
Source: Wellcome Open Res. 2020 Jun 4;4:79. Originally published 2019 May 10. [Version 4] doi: 10.12688/wellcomeopenres.15217.4 (PMC7323594; doi:10.12688/wellcomeopenres.15217.4)
Supplement: Peer review history

# Peer review: Reviewer response for version 4

**DOI:** 10.21956/wellcomeopenres.17530.r38968

**Published:** 2020-06-26

**Authors:** Nicola Low

**Affiliations:** 1 Institute of Social and Preventive Medicine, University of Bern, Bern, Switzerland

The authors have replied carefully and completely to all my comments. I am happy to approve indexing.

Is the work clearly and accurately presented and does it cite the current literature?

Partly

If applicable, is the statistical analysis and its interpretation appropriate?

Partly

Are all the source data underlying the results available to ensure full reproducibility?

Yes

Is the study design appropriate and is the work technically sound?

Partly

Are the conclusions drawn adequately supported by the results?

Partly

Are sufficient details of methods and analysis provided to allow replication by others?

Partly

Reviewer Expertise:

Epidemiology, bacterial sexually transmitted infections, antimicrobial resistance.

I confirm that I have read this submission and believe that I have an appropriate level of expertise to confirm that it is of an acceptable scientific standard.

# Peer review: Reviewer response for version 3

**DOI:** 10.21956/wellcomeopenres.17260.r37970

**Published:** 2020-02-24

**Authors:** Nicola Low

**Affiliations:** 1 Institute of Social and Preventive Medicine, University of Bern, Bern, Switzerland

Thank you for giving me the opportunity to review the revised version of this manuscript again. The revisions address my previous comments, except for the reporting of antimicrobial resistance. I apologise if these comments appear picky, but they are important for interpretation of the data presented. 

1. Methods (p4, column 1):
  - the authors say disc diffusion was used to assess antimicrobial susceptibility to penicillin. The authors confirm that the results are MICs, ranging from 32 to 0.024. I looked at the US CDC website as I thought this should be up to date ( https://www.cdc.gov/std/gonorrhea/lab/diskdiff.htm). According to these interpretive criteria for disc diffusion (using CLSI values), there are three categories of size of inhibition zone, corresponding with three categories of MIC. Can the authors explain how they were able to define MICs so precisely?
  - The authors give a CLSI cut-off for cefixime of ≤0.5µg/ml as indicating susceptibility. The citation (ref 29) is dated 2017. It is possible that these cut-offs have been revised. The online CDC document (citing CLSI) gives ≤0.25µg/ml as indicating susceptibility. Please clarify. 
2. Results (p6, column 1):
  - Please delete 100%, this is unnecessary as it says “All… were resistant…”
  - I don’t understand the sentence, “Of the 6, 2 were partially resistant and 4 had intermediate susceptibility to penicillin.” According to Table 3, and CLSI isolates 1 and 2 are resistant (MIC 32 and 8, respectively); isolates 3, 4 and 5 are intermediate (MIC 0.19, 0.25, 0.19, respectively); isolate 6 is susceptible (MIC 0.024). Please clarify.
  - Table 3: the second footnote should be split – the footnote with * is on the same line.
  - Table 3: the order of the footnotes is in a different order to the columns. This is a bit confusing, since the name of the antibiotic isn’t given, and some values are quite similar. Could the authors re-order, and use either superscripts in alphabetical order or standard symbols, e.g. https://en.wikipedia.org/wiki/Note_(typography)?

Is the work clearly and accurately presented and does it cite the current literature?

Partly

If applicable, is the statistical analysis and its interpretation appropriate?

Partly

Are all the source data underlying the results available to ensure full reproducibility?

Yes

Is the study design appropriate and is the work technically sound?

Partly

Are the conclusions drawn adequately supported by the results?

Partly

Are sufficient details of methods and analysis provided to allow replication by others?

Partly

Reviewer Expertise:

Epidemiology, bacterial sexually transmitted infections, antimicrobial resistance.

I confirm that I have read this submission and believe that I have an appropriate level of expertise to confirm that it is of an acceptable scientific standard, however I have significant reservations, as outlined above.

# Response

**Published:** 2020-05-15

**Authors:** Caroline Ngetsa

**Affiliations:** KWTRP, Kenya

Dear Reviewer,

Many thanks for your comments on our manuscript. We have responded to each comment and include the changes in bold font in the amended manuscript.

1. *Methods (p4, column 1):*

- *The authors say disc diffusion was used to assess antimicrobial susceptibility to penicillin. The authors confirm that the results are MICs, ranging from 32 to 0.024. I looked at the US CDC website as I thought this should be up to date ( https://www.cdc.gov/std/gonorrhea/lab/diskdiff.htm). According to these interpretive criteria for disc diffusion (using CLSI values), there are three categories of size of inhibition zone, corresponding with three categories of MIC. Can the authors explain how they were able to define MICs so precisely?*

*The authors give a CLSI cut-off for cefixime of ≤0.5µg/ml as indicating susceptibility. The citation (ref 29) is dated 2017. It is possible that these cut-offs have been revised. The online CDC document (citing CLSI) gives ≤0.25µg/ml as indicating susceptibility. Please clarify.*

In our revision, we have added to the methodology the MIC breakpoints for penicillin (in bold font in the methods text below). In addition, we added a column for penicillin disc diffusion in mm and reordered the columns of table 3. The new Table 3 groups disc diffusion results for penicillin, ceftriaxone, and tetracycline and E-test results for penicillin, cefixime, and ciprofloxacin. The revised table 3 is included below.

We have rectified the revised manuscript  with additional text in bold font, as follows: “The disc diffusion test was used to assess antimicrobial susceptibility of NG to ceftriaxone, penicillin and tetracycline ^30^ . The E-test was performed to obtain minimum inhibitory concentrations (MIC) for cefixime, ciprofloxacin **and penicillin**; cut-off values were obtained from the Clinical and Laboratory Standards Institute (CLSI) ^29^ .

For cefixime, a MIC **≤0.25** μg/ml indicated susceptibility. For ciprofloxacin, a MIC of ≤0.06 μg/ml indicated susceptibility, 0.12-0.5 μg/ml intermediate susceptibility, and ≥1 μg/ml resistance **while for penicillin, a MIC of ≤0.06 μg/ml indicated susceptibility, 0.12-1.0 μg/ml intermediate susceptibility, and ≥2 μg/ml resistance.”**

**Revised Table 3 “Antimicrobial susceptibility of rectal *Neisseria gonorrhoeae* isolates”:**

**Disc diffusion (Inhibitory zone in mm)**

**E Test (MIC in µg/ml)**

**Isolate**

**Penicillin ^a^**

**Ceftriaxone ^b^**

**Tetracycline ^c^**

**Penicillin ^d^**

**Cefixime ^e^**

**Ciprofloxacin ^f^**

**1**

12

45

14

32

0.016

4

**2**

6

36

18

8

0.016

3

**3**

39

45

13

0.19

0.016

16

**4**

34

40

14

0.25

0.016

4

**5**

38

45

19

0.19

0.016

2

**6**

37

45

16

0.024

0.016

4

^a: ^ Zone ≥47mm indicates susceptibility, 27- 46 mm indicates intermediate    susceptibility and ≤26mm resistance.

^b: ^Zone ≥35mm indicates susceptibility. 

^c: ^Zone ≥38mm indicates susceptibility, 31–37mm intermediate susceptibility, ≤30mm resistance.

^d: ^MIC ≤0.06µg/ml indicates susceptibility, 0.12-1 µg/ml intermediate susceptibility and ≥2µg/ml resistance.

^e: ^MIC ≤0.25µg/ml indicates susceptibility 

^f: ^MIC of ≤0.06µg/ml indicates susceptibility, 0.12-0.5µg/ml intermediate susceptibility, and ≥1µg/ml resistance

*2. Results (p6, column 1):*

- *Please delete 100%, this is unnecessary as it says “All… were resistant…”*

Thank you for the comment, the 100% has been deleted.

*I don’t understand the sentence, “Of the 6, 2 were partially resistant and 4 had intermediate susceptibility to penicillin.” According to Table 3, and CLSI isolates 1 and 2 are resistant (MIC 32 and 8, respectively); isolates 3, 4 and 5 are intermediate (MIC 0.19, 0.25, 0.19, respectively); isolate 6 is susceptible (MIC 0.024). Please clarify.*

Thank you for the observation, the reviewer is correct. We have rectified the revised manuscript to read ‘ **Of the 6, 2 were resistant, 3 had intermediate susceptibility to penicillin, and 1 was susceptible to penicillin. **

- Table 3: the second footnote should be split – the footnote with * is on the same line.

Thank you for the observation, we have corrected and rearranged all footnotes in the manuscript (i.e. in the new table 3) in the order of drug listed in table 3.

*Table 3: the order of the footnotes is in a different order to the columns. This is a bit confusing, since the name of the antibiotic isn’t given, and some values are quite similar. Could the authors re-order, and use either superscripts in alphabetical order or standard symbols, e.g. https://en.wikipedia.org/wiki/Note_(typography)?*   

Thank you for the observation, we have corrected the order of the footnotes and have replaced symbols with superscripts in alphabetical as shown in Table 3 above.

We would like to thank you for making these excellent review points helping us to improve communication about our work. We trust that we have addressed these additional points and hope that our manuscript will meet your approval.

Yours sincerely,

Caroline Ngetsa

# Peer review: Reviewer response for version 2

**DOI:** 10.21956/wellcomeopenres.16995.r37444

**Published:** 2019-12-27

**Authors:** Nicola Low

**Affiliations:** 1 Institute of Social and Preventive Medicine, University of Bern, Bern, Switzerland

Thank you for giving me the opportunity to review the revised version of this manuscript. The authors have done a good job of responding to almost all of my comments. There are a couple of outstanding issues, which I hope the authors will be able to address. 

1. Reporting of response rate and comparison between responders and non-responders: thank you for describing the enrolment more completely. Unfortunately, the main text and the response are now contradictory. The main text says that 104 men were enrolled out of a possible 174 (60%). In the response, the authors say that “We were able to enrol 70 of the 174 eligible men (i.e. 40%). We have added a supplemental table…” The supplementary material is the Stata output and the definitions of the value labels for participation (0 or 1) aren’t given. It would help to summarise the Stata output in a table, with “Enrolled” or “Not enrolled”.
2. Antimicrobial treatment: please can you say which guidelines you followed for treatment (are they Kenyan guidelines) because the treatment given, whilst likely to be effective, is not the same as the WHO recommendations, which you cite (your refs 22 and 23).
3. Antimicrobial resistance: thank you for tabulating the findings of antimicrobial susceptibility testing. This is much more useful. Could you please now clarify the interpretation of the disc diffusion results:
  - The ref you give, Liu H et al. is for cephalosporins only. That paper says an inhibitory zone of >=35mm is susceptible – can you add that this is the cut-off?
  - The penicillin values look, to me, more like MIC value, e.g. ranging from 32 to 0.024 – how could 0.024 be read in mm? Please clarify.
  - Please give a ref and a cut-off for tetracycline susceptibility.
4. Data analysis and Table 1: I requested global p-values from a likelihood ratio test. I understand your reasons for wanting to present stratum specific results. My understanding is that, in the multivariable model, you want to compares the model fit for the whole variable. The likelihood ratio test gives this information. You can then show, using the point estimate and its confidence interval that the adjusted OR in one stratum is greater, or smaller, than the reference category. I am not going to insist, but in any case, please remove the bold for p-values. It is unnecessary.
5. Abstract – you could remove the numbers about prevalence at follow up. It would be more useful to give the confidence intervals for the prevalences that you state.

Is the work clearly and accurately presented and does it cite the current literature?

Partly

If applicable, is the statistical analysis and its interpretation appropriate?

Partly

Are all the source data underlying the results available to ensure full reproducibility?

Yes

Is the study design appropriate and is the work technically sound?

Partly

Are the conclusions drawn adequately supported by the results?

Partly

Are sufficient details of methods and analysis provided to allow replication by others?

Partly

Reviewer Expertise:

Epidemiology, bacterial sexually transmitted infections, antimicrobial resistance.

I confirm that I have read this submission and believe that I have an appropriate level of expertise to confirm that it is of an acceptable scientific standard, however I have significant reservations, as outlined above.

# Response

**Published:** 2020-02-13

**Authors:** Caroline Ngetsa

**Affiliations:** KWTRP, Kenya

Dear Reviewer,

Many thanks for your careful review of our revised manuscript and alerting us to few outstanding issues. Below, we have responded to your comments and indicated changes that we requested to be made to the manuscript.

- *Reporting of response rate and comparison between responders and non-responders: thank you for describing the enrolment more completely. Unfortunately, the main text and the response are now contradictory. The main text says that 104 men were enrolled out of a possible 174 (60%). In the response, the authors say that “We were able to enrol 70 of the 174 eligible men (i.e. 40%). We have added a supplemental table…” *

**There was an error in our response letter. We confirm that the manuscript text is correct. The supplemental table also presented the correct numbers.**

*The supplementary material is the Stata output and the definitions of the value labels for participation (0 or 1) aren’t given. It would help to summarise the Stata output in a table, with “Enrolled” or “Not enrolled”.*

**We have changed the labels for participation (0 or 1) to (Not enrolled or Enrolled) on the supplemental table and the reference 45 has also been updated with these corrections.**

- *Antimicrobial treatment: please can you say which guidelines you followed for treatment (are they Kenyan guidelines) because the treatment given, whilst likely to be effective, is not the same as the WHO recommendations, which you cite (your refs 22 and 23).*

**Thank you for picking up on this omission. Our STD treatment followed from a 2015 Kenyan guidelines revision that initially proposed 2 gram azithromycin stat. The Kenyan guidelines have since aligned with WHO guidelines and changed to 1 gram azithromycin stat. We erroneously reported the old treatment regimen but have corrected it now in the manuscript (1 gram azithromycin as per Kenyan and WHO guidelines). **

- *Antimicrobial resistance: thank you for tabulating the findings of antimicrobial susceptibility testing. This is much more useful. Could you please now clarify the interpretation of the disc diffusion results*:

**We have updated the table with the antimicrobial sensitivity results with interpretation for both the disc diffusion and MIC results.**

- *The ref you give, Liu H et al. is for cephalosporins only. That paper says an inhibitory zone of >=35mm is susceptible – can you add that this is the cut-off*

**We have added the cephalosporin cut off >=35mm in the table. We have also included cut offs for the other drugs listed in the table.**

- *The penicillin values look, to me, more like MIC value, e.g. ranging from 32 to 0.024 – how could 0.024 be read in mm? Please clarify*.

**We acknowledge that this was an error in the column label. We have updated the column label to indicate ‘MIC in** **µg/ml’**

- *Please give a ref and a cut-off for tetracycline susceptibility.*

**Ref: Performance Standards for antimicrobial susceptibility test; CLSI 2019 guidelines. Cut-off for Tetracycline >=38mm is susceptible.**

- *Data analysis and Table 1: I requested global p-values from a likelihood ratio test. I understand your reasons for wanting to present stratum specific results. My understanding is that, in the multivariable model, you want to compare the model fit for the whole variable. The likelihood ratio test gives this information. You can then show, using the point estimate and its confidence interval that the adjusted OR in one stratum is greater, or smaller, than the reference category. I am not going to insist, but in any case, please remove the bold for p-values. It is unnecessary*.
- *Abstract – you could remove the numbers about prevalence at follow up. It would be more useful to give the confidence intervals for the prevalences that you state.*

**Thank you for your consideration with regards to Table 1, and the suggestion to remove the prevalences at time point 2 in the abstract. We consider removing the bolding of p-values a minor edit and proposed abstract edits of minorly importance and prefer to leave this as is.**

We would like to thank you for making these excellent review points helping us to improve communication about our work. We trust that we have addressed these additional points and hope that our manuscript will meet your approval.

# Peer review: Reviewer response for version 1

**DOI:** 10.21956/wellcomeopenres.16606.r36001

**Published:** 2019-08-13

**Authors:** Nicola Low

**Affiliations:** 1 Institute of Social and Preventive Medicine, University of Bern, Bern, Switzerland

Thank you for giving me the opportunity to review this manuscript, which addresses the important issue of extra-genital sexually transmitted infections (STI) in men who have sex with men (MSM) in Kenya. I have no concerns about the relevance and validity of the data. The study is small but, on the other hand, has detailed behavioural information, antimicrobial susceptibility data and a follow-up visit. The findings lack statistical precision, so the authors are restricted in the certainty of the conclusions that they can draw. In this situation, data from such studies are very important for future syntheses of evidence in systematic reviews.

 In Wellcome Open Research, I think the authors have more flexibility about what to report, meaning that there is an opportunity for useful contextual information about the study and for full reporting that will make the findings reproducible and able to be used in a future systematic review. I have some suggestions that I hope the authors will find useful in revising their manuscript.  

**Major comments**

1. Study design: this is a report of a cohort study, nested within a larger cohort study. Please make sure that all the items proposed in the STROBE (Strengthening the Reporting of Observational studies in Epidemiology) checklist for cohort studies ( https://www.strobe-statement.org/index.php?id=available-checklists) are reported. In particular, I would like to see: a) the proportion enrolled and whether or not they differed from the men in the larger cohort study. The authors only report (p4, Results, Participant backgrounds, line 1), “104 participants were recruited and tested”; b) the follow-up time reported as a total number of months and average for each participant; 95% confidence intervals (CI) for the main percentages.
2. Study design: Can the authors say whether this study was pre-planned? If so, was there a target sample size with a power calculation? Was enrolment less than expected?
3. Data analysis: can the authors please calculate and report the incidence rate of *Chlamydia trachomatis *(CT) and *Neisseria gonorrhoeae* (NG) detection in anorectal samples? The availability of data from a follow-up visit is a strength of the study. Prevalence at follow-up (p4, Methods, Data analysis, line 3, and Table 2) isn’t very useful. It would be great to change the columns in Table 2 to report: numbers with positive results at baseline; the numbers of incident cases and follow-up time; incidence rate (with 95% CI) in those with a negative test result at baseline; repeat detection rate (with 95% CI) in those with a positive test at baseline who received treatment.
4. Data analysis (p4, Methods, Data analysis, line 11-13): Please delete the sentence with “…a cut-off p-value of p<0.05…” The p-value will be driven by the sample size and not clinical relevance. There are no pre-specified hypotheses, so null hypothesis significance testing does not make sense.
5. Antimicrobial resistance: thank you for reporting results of antimicrobial susceptibility testing even though culture at baseline was unsuccessful. The recovery rate at follow-up (6 of 12 GeneXpert positive results is as expected for rectal swabs). I didn’t find the presentation of the data in Figure 3 or percentages in the text very useful. A table of the results for each isolate would take up the same amount of space as Figure 3 and, for each antimicrobial tested, the MIC for each isolate could be shown. In the text, the absolute numbers are more useful than the percentages.

**Minor comments**

1. The causative agent of gonorrhoea is *Neisseria gonorrhoeae *(not *gonorrhoea*). Please check the spelling throughout.
2. “Burden” – this term is used several times to refer to the frequency of infection. “Burden of disease” usually includes a measurement of the consequences of the condition, e.g. a disability-adjusted life year. With asymptomatic infections for which the clinical complications are unknown or rare, burden is not really an appropriate term.
3. Introduction (p3. Para 1): Literature review. It would be useful to be a bit more precise in the reporting of the chosen studies:
  - Of interest, you may wish to update ref 2. WHO has just published its newest estimates for 2016 ( https://www.who.int/bulletin/volumes/97/8/18-228486.pdf). The estimates of global prevalence for CT and NG for men are v similar to 2012. For men in the African region, 2016 estimates for urogenital infection in men are: CT 4.0% (95% CI 2.4–6.1), NG 1.6% (95% CI 0.9–2.6).
  - Line 8, “Worldwide” is not really accurate – these are two studies, one in Italy and one in Thailand is particularly high risk populations. Please rephrase.
  - Differences in reports of prevalence will differ depending on whether the study population is unselected or all report recent receptive anal intercourse. Your description should take these factors into account.
  - Is there a systematic review? If so, please cite. If not, maybe worth pointing out in Discussion as a research need.
4. Introduction (p3, ref 10): you could acknowledge that this estimate comes from your own group.
5. Introduction (p3, para 2): “…(NAAT)… has superseded the use of cultures…” NAAT are not available everywhere so you may want to qualify this statement with “where available”.
6. Methods, Sample collection (p4): Could you give more detail about the “standardized methodology” for collection of the swabs? Was a proctoscope used? How far inside the anal canal was the swab inserted? Was it moistened? These are important details for reproducibility. Technically, the swab is a rectal swab only if inserted past the dentate line, so swabs collected without proctoscopy, which sample mainly from the anal canal might be more accurately termed as “anorectal”.
7. Data analysis (p4, Methods, line 4): “bivariable” is ambiguous. When the model examines an association between an outcome and one other variable, it is usual to call this a univariable analysis.
8. Data analysis and Table 1: the p-values are given for each stratum (e.g. age, religion). In the multivariable statistical model, it is the contribution of the variable as a whole that is important. These ‘global’ p values should be derived from a likelihood ratio test that compares the model fit with the variable to the model that includes all variables other than the one of interest. For a binary variable the results are the same.
9. Table 1: is the ethnic origin of MSM in Kenya associated with their risk of rectal STIs? If so, ethnic group might be a useful descriptive variable to report.
10. Discussion: This could be better structured. It is important that you collected data about infections, behaviours and antimicrobial susceptibility. Both presumptive treatment and frequent screening and treatment impose selection pressure for the emergence of antimicrobial resistance. The discussion is an opportunity to bring together these lines of thought in thinking about how to address rectal STIs in MSM.
11. Discussion, Limitations (p7): It’s worth mentioning the limitation of having combined the data for CT and NG owing to the small sample size.
12. Conclusion (p7): “…between 21.2% and 25%.” These point estimates suggest spurious precision and lower 95% CI is much lower. I expect each estimate lies within the confidence interval of the other, given the sample size. Please rephrase in context of confidence intervals.
13. Conclusions: in Abstract (p1, and main text (p7) don’t match. In the Abstract, there is no support from the results that “The high prevalence of asymptomatic rectal CT and NG in MSM reporting RAI demonstrates the need for frequent screening”. The more cautious statements in the main text are more appropriate.
14. Dataset: could you add a worksheet with a key to the abbreviations for the variables?

Is the work clearly and accurately presented and does it cite the current literature?

Partly

If applicable, is the statistical analysis and its interpretation appropriate?

Partly

Are all the source data underlying the results available to ensure full reproducibility?

Yes

Is the study design appropriate and is the work technically sound?

Partly

Are the conclusions drawn adequately supported by the results?

Partly

Are sufficient details of methods and analysis provided to allow replication by others?

Partly

Reviewer Expertise:

Epidemiology, bacterial sexually transmitted infections, antimicrobial resistance.

I confirm that I have read this submission and believe that I have an appropriate level of expertise to confirm that it is of an acceptable scientific standard, however I have significant reservations, as outlined above.

# Response

**Published:** 2019-12-17

**Authors:** Caroline Ngetsa

**Affiliations:** KWTRP, Kenya

Dear Reviewers,

Many thanks for your comments on our manuscript. We have responded to each individually and reflect the changes in the amended manuscript.

*Major comments*

*Study design: this is a report of a cohort study, nested within a larger cohort study. Please make sure that all the items proposed in the STROBE (Strengthening the Reporting of Observational studies in Epidemiology) checklist for cohort studies (* *https://www.strobe-statement.org/index.php?id=available-checklists* *) are reported.*

**Thank you for your review. We have ensured that all elements of the STROBE checklist are included, and have responded to each of your comments individually. We will make sure this is visible in the revised paper.**

*In particular, I would like to see: a) the proportion enrolled and whether or not they differed from the men in the larger cohort study. The authors only report (p4, Results, Participant backgrounds, line 1), “104 participants were recruited and tested”;*

**Between April 2016 and January 2017, eligible participants were identified at follow-up visits in an existing cohort study on HIV acquisition, based on self-report of any receptive anal intercourse (RAI) within the past 6 months. We were able to enrol 70 of 174 eligible men (i.e. 40%). We** **have added a supplemental table that can be accessed on *figshare* (link in References section) to compare characteristics of the 104 cohort participants not included with those of the 70 participants included in this study: “** **Participants not enrolled were more likely to report sex with men exclusively, transactional sex, RAI, group sex, and any unprotected sex (data in supplemental material).**

*b) the follow-up time reported as a total number of months and average for each participant; 95% confidence intervals (CI) for the main percentages.*

**Total follow-up was 5.7 months on average (range 5.4-6.2 months) for each participant, which amounted to a total follow-up time of 39.6 person-years.**

*Study design: Can the authors say whether this study was pre-planned? If so, was there a target sample size with a power calculation? Was enrolment less than expected?*

**This study was planned and done to estimate prevalence and incidence of CT/NG infections among MSM participants who reported RAI. We did not have a prespecified hypothesis, therefore a sample size was not calculated. The precision estimate of the confidence interval was based on exact binomial confidence intervals. The study was limited by a small budget to conduct the study.**

*Data analysis: can the authors please calculate and report the incidence rate of Chlamydia trachomatis (CT) and Neisseria gonorrhoeae (NG) detection in anorectal samples? The availability of data from a follow-up visit is a strength of the study. Prevalence at follow-up (p4, Methods, Data analysis, line 3, and Table 2) isn’t very useful.*

*It would be great to change the columns in Table 2 to report: numbers with positive results at baseline; the numbers of incident cases and follow-up time; incidence rate (with 95% CI) in those with a negative test result at baseline; repeat detection rate (with 95% CI) in those with a positive test at baseline who received treatment.*

**Thank you for the suggestion. Given that the data is available, we have used it to calculate incidence as well and revised our Table 2 to reflect this.**

*Data analysis (p4, Methods, Data analysis, line 11-13): Please delete the sentence with “…a cut-off p-value of p<0.05…” The p-value will be driven by the sample size and not clinical relevance. There are no pre-specified hypotheses, so null hypothesis significance testing does not make sense.*

**Thank you for highlighting this.**

*Antimicrobial resistance: thank you for reporting results of antimicrobial susceptibility testing even though culture at baseline was unsuccessful. The recovery rate at follow-up (6 of 12 GeneXpert positive results is as expected for rectal swabs). I didn’t find the presentation of the data in Figure 3 or percentages in the text very useful. A table of the results for each isolate would take up the same amount of space as Figure 3 and, for each antimicrobial tested, the MIC for each isolate could be shown. In the text, the absolute numbers are more useful than the percentages.*

**Many thanks for these comments. We have included a Table 3 instead of Figure 1, which presents the data you have suggested. In addition, we have written down whole numbers instead of percentages when describing the AMR data in the manuscript.**

*Minor comments*

*The causative agent of gonorrhoea is Neisseria gonorrhoeae (not gonorrhoea). Please check the spelling throughout.*

**Thank you for highlighting this.**

*“Burden” – this term is used several times to refer to the frequency of infection. “Burden of disease” usually includes a measurement of the consequences of the condition, e.g. a disability-adjusted life year. With asymptomatic infections for which the clinical complications are unknown or rare, burden is not really an appropriate term.*

**We have rethought our use of this word and ensured appropriate terminology is used throughout this manuscript.**

*Introduction (p3. Para 1): Literature review. It would be useful to be a bit more precise in the reporting of the chosen studies:*

*Of interest, you may wish to update ref 2. WHO has just published its newest estimates for 2016 (* *https://www.who.int/bulletin/volumes/97/8/18-228486.pdf* *). The estimates of global prevalence for CT and NG for men are v similar to 2012. For men in the African region, 2016 estimates for urogenital infection in men are: CT 4.0% (95% CI 2.4–6.1), NG 1.6% (95% CI 0.9–2.6).*

**Thank you for pointing us to this updated piece of literature, we have updated our manuscript to reflect the most recent evidence.**

*Line 8, “Worldwide” is not really accurate – these are two studies, one in Italy and one in Thailand is particularly high risk populations. Please rephrase.*

**Thank you, we have been more specific in describing the results found.**

*Differences in reports of prevalence will differ depending on whether the study population is unselected or all report recent receptive anal intercourse. Your description should take these factors into account.*

*Is there a systematic review? If so, please cite. If not, maybe worth pointing out in Discussion as a research need.*

**Many thanks for pointing out that it may seem that way. It was not our intention to do a systematic review, but rather just present existing estimates of rectal STIs in MSM in other geographical regions than Kenya. This serves as a prelude to presenting our own data. We have included in our limitations the fact that we did not systematically review all existing literature.**

*Introduction (p3, ref 10): you could acknowledge that this estimate comes from your own group.*

**Thank you, we have now acknowledged our own results.**

*Introduction (p3, para 2): “…(NAAT)… has superseded the use of cultures…” NAAT are not available everywhere so you may want to qualify this statement with “where available”.*

**This has been amended.**

*Methods, Sample collection (p4): Could you give more detail about the “standardized methodology” for collection of the swabs? Was a proctoscope used? How far inside the anal canal was the swab inserted? Was it moistened? These are important details for reproducibility. Technically, the swab is a rectal swab only if inserted past the dentate line, so swabs collected without proctoscopy, which sample mainly from the anal canal might be more accurately termed as “anorectal”.*

**This has been detailed in the revised manuscript. We used proctoscopy and dry swab kits for sample collection.**

*Data analysis (p4, Methods, line 4): “bivariable” is ambiguous. When the model examines an association between an outcome and one other variable, it is usual to call this a univariable analysis.*

**Thank you for pointing this out, we have amended this.**

*Data analysis and Table 1: the p-values are given for each stratum (e.g. age, religion). In the multivariable statistical model, it is the contribution of the variable as a whole that is important. These ‘global’ p values should be derived from a likelihood ratio test that compares the model fit with the variable to the model that includes all variables other than the one of interest. For a binary variable the results are the same.*

**Thank you for this suggestion. When we apply the p-values derived from the likelihood ratio test, we lose individual characteristic of the variable with more than 2 strata. For example, formal employment was associated with prevalent infection (borderline) when we apply the Wald p-values. When we apply the likelihood ratio test, this association becomes concealed despite having an estimate with CI’s above 0. We therefore prefer to present the Wald P values for this analysis.**

*Table 1: is the ethnic origin of MSM in Kenya associated with their risk of rectal STIs? If so, ethnic group might be a useful descriptive variable to report.*

**Thank you, we looked at this value as 5 major ethnic categories and there is no significant difference by ethnicity.**

*Discussion: This could be better structured. It is important that you collected data about infections, behaviours and antimicrobial susceptibility. Both presumptive treatment and frequent screening and treatment impose selection pressure for the emergence of antimicrobial resistance. The discussion is an opportunity to bring together these lines of thought in thinking about how to address rectal STIs in MSM.*

**Many thanks for pointing this out. We have now better structured our conclusion and highlighted the very important fact that these methods could increase AMR.**

*Discussion, Limitations (p7): It’s worth mentioning the limitation of having combined the data for CT and NG owing to the small sample size.*

**Thank you for your suggestion, we have now added this as one of our limitations.**

*Conclusion (p7): “…between 21.2% and 25%.” These point estimates suggest spurious precision and lower 95% CI is much lower. I expect each estimate lies within the confidence interval of the other, given the sample size. Please rephrase in context of confidence intervals.*

**Thank you, we’ve done this now.**

*Conclusions: in Abstract (p1, and main text (p7) don’t match. In the Abstract, there is no support from the results that “The high prevalence of asymptomatic rectal CT and NG in MSM reporting RAI demonstrates the need for frequent screening”. The more cautious statements in the main text are more appropriate.*

**We have modified the abstract to reflect this, thank you.**

*Dataset: could you add a worksheet with a key to the abbreviations for the variables?*

**We have now included a worksheet with dataset abbreviations (it can be found on *figshare*, and there is a link to that in the References section of the manuscript).**

# Peer review: Reviewer response for version 1

**DOI:** 10.21956/wellcomeopenres.16606.r35502

**Published:** 2019-05-28

**Authors:** Michael W. Ross

**Affiliations:** 1 Program in Human Sexuality, Department of Family Medicine, University of Minnesota Medical School, Minneapolis, MN, USA

This is a useful report of rectal gonorrhea and chlamydia in MSM in coastal Kenya. The Introduction suffers from missing some crucial findings from a similar study of prevalence of rectal NG/CT in MSM in Tanga and Dar es Salaam, a few hundred miles south of where this study was carried out (Ross MW *et al*., BMJ Open 2014) ^1^. Some of those interesting findings were replicated, including the lack of association between HIV and STI in MSM, plus similar high rates of rectal STIs. There may be lessons here about access to clinics that will perform rectal examinations/swabs, especially if the MSM are not "out" to health care workers (rectal STIs are a marker for MSM activity and likely to engender discriminatory behavior in many health workers). Religion was given for 77%, but what was the other quarter? Hindi? No religion? 

 This is an important study, with the gaps noted above. It would also have been helpful to briefly speculate as to what bias might have been associated with recruiting participants from an existing high-risk cohort study.

Is the work clearly and accurately presented and does it cite the current literature?

Partly

If applicable, is the statistical analysis and its interpretation appropriate?

Yes

Are all the source data underlying the results available to ensure full reproducibility?

Yes

Is the study design appropriate and is the work technically sound?

Yes

Are the conclusions drawn adequately supported by the results?

Yes

Are sufficient details of methods and analysis provided to allow replication by others?

Yes

Reviewer Expertise:

Behavioral Epidemiology of STIs and HIV in MSM, Africa.

I confirm that I have read this submission and believe that I have an appropriate level of expertise to confirm that it is of an acceptable scientific standard.

## References

[1] : High HIV seroprevalence, rectal STIs and risky sexual behaviour in men who have sex with men in Dar es Salaam and Tanga, Tanzania. *BMJ Open*.2014;4(8) : 10.1136/bmjopen-2014-006175 e006175 10.1136/bmjopen-2014-006175 25168042PMC4156794

# Response

**Published:** 2019-12-17

**Authors:** Caroline Ngetsa

**Affiliations:** KWTRP, Kenya

Dear Reviewers,

Many thanks for your comments on our manuscript. We have responded to each individually and reflect the changes in the amended manuscript.

*This is a useful report of rectal gonorrhea and chlamydia in MSM in coastal Kenya. The Introduction suffers from missing some crucial findings from a similar study of prevalence of rectal NG/CT in MSM in Tanga and Dar es Salaam, a few hundred miles south of where this study was carried out (Ross MW et al., BMJ Open 2014)* *1* *. Some of those interesting findings were replicated, including the lack of association between HIV and STI in MSM, plus similar high rates of rectal STIs. There may be lessons here about access to clinics that will perform rectal examinations/swabs, especially if the MSM are not "out" to health care workers (rectal STIs are a marker for MSM activity and likely to engender discriminatory behavior in many health workers). Religion was given for 77%, but what was the other quarter? Hindi? No religion?*

**Thank you for your review. We have now referred to the study in Dar Es Salaam and Tanga in the background and discussion sections of this manuscript. The point of discussion regarding access to clinics is also very relevant; we feel that we do raise this issue a bit in our introduction and discussion, by raising issues with stigma related to MSM in general; this should encompass healthcare access as well.**

**We have also written down the religious background of the remaining quarter of participants; this is also visible in Table 1.**

*This is an important study, with the gaps noted above. It would also have been helpful to briefly speculate as to what bias might have been associated with recruiting participants from an existing high-risk cohort study.*

**Thank you for noting this. We have included details of eligible patients who were not included in the study (and have included data available on *figshare*, a link is available in the References of the manuscript): importantly, it was noted that patients not included in the study had higher risk behaviour. This is unlikely to underestimate true infection prevalence and rate within this population.**
